# Perspective: rethinking therapeutic strategies in oncology

**DOI:** 10.3389/fonc.2023.1335987

**Published:** 2024-01-10

**Authors:** Edward F. Patz, Elizabeth B. Gottlin, George R. Simon

**Affiliations:** ^1^Department of Radiology, Duke University School of Medicine, Durham, NC, United States; ^2^Department of Pharmacology and Cancer Biology, Duke University School of Medicine, Durham, NC, United States; ^3^Department of Medical Oncology at Advent Health, Moffitt Cancer Center, Tampa, FL, United States

**Keywords:** immuno-oncology, tumor microenvironment, immunosuppression, oncology clinical trials, response evaluation

## Abstract

Immuno-oncology has revolutionized cancer care, drug development, the design of clinical trials, standard treatment paradigms, and the evaluation of response to therapy. These are all areas, however, that have not fully incorporated principles of tumor immunology. Insufficient emphasis is put on the effect drugs have on the immune system, and specifically, the impact that multiple lines of therapy can have on the functioning of the immune system, hindering a robust anti-tumor immune response. A paradigm shift in how we approach the development of novel immunotherapeutic agents is necessary to facilitate the effective improvements in patient outcomes.

## Introduction

Cancer is a diverse and complex genetically driven disease, and is a major worldwide public health problem. The development of cancer is not an arbitrary or capricious process but a series of well-orchestrated events that result in a convergent and regressive phenotype. Tumor cells acquire fundamental traits of an invasive species: rapid reproduction, high dispersal ability, loss of primary function, and phenotypic plasticity (the ability to grow in a wide range of different environmental conditions). However, tumor cells exist in both a micro and macro environment and the interaction between tumor cells and the host is intimately tied to outcome. Aside from immuno-oncology regimens that to date primarily focus on cytotoxic T cells and help only a subset of patients, current cancer therapy does not fully take advantage of this tumor-host interaction: Paradigm shifts are long overdue in the areas of drug development, clinical guidelines, clinical trial design, and metrics to gauge response, based on immunological considerations.

## Anti-tumor immunity: the key to successful cancer therapy

It has become apparent that the fundamental unifying principle for durable long-term survival is the generation of effective anti-tumor immunity. Although this concept is well described, it has not been integrated effectively into basic science investigations, clinical investigations or clinical practice. There are a variety of strategies that attempt to achieve such integration, but with many different stakeholders including scientists, clinicians, and pharmaceutical companies, each having their own perspective and expertise, it is difficult to change the approaches that have led therapeutic investigations for decades. Much of the complex biology is lost in the search for the next best target and drug, and only incremental advances are realized. Furthermore, clinical guidelines for patient management and response assessment are stagnant. The quantum changes that are needed in cancer therapy require patience, persistence, and substantial resources to disrupt current practice. While immuno-oncology has had a significant impact on cancer therapy, it can cause considerable toxicity. This perspective outlines some of the challenges and opportunities for developing better therapeutic strategies that focus on anti-tumor immunity in order to improve outcomes.

The host response is vital to outcomes. Studies in a variety of tumor types have shown an association between intratumoral effector T lymphocytes and an improved prognosis. Furthermore, the presence of intratumoral tertiary lymphoid structures, in which B and T cells interact to promote antibody affinity maturation and class switching, are also associated with a better prognosis (1). Conversely, tumors in which immunosuppressive cell types such as monocyte-derived suppressor cells, T regulatory cells, M2 macrophages, and N2 neutrophils predominate are associated with a negative prognosis and resistance to immune checkpoint therapy (2). An ideal cancer therapy is likely to be one that both kills cancer cells directly and switches the tumor microenvironment from one that promotes to one that inhibits cancer growth.

In addition to the intratumoral immune response, clinical observations such as the abscopal effect, in which distant sites of metastasis regress following local radiotherapy, are suggestive of a systemic anti-tumor immune response (3, 4). However, in a variety of human cancers, changes in the host periphery caused by tumor burden include expansion of immunosuppressive cells of myeloid origin (e.g., neutrophils and monocytes) as well as T regulatory cells; decrease in dendritic cell subsets needed to activate effector T cells; and reduction in T cell functionality and overall diversity of T cell receptors; a detailed summary of specific immune related changes in cancer are presented in a recent review (5). Chemotherapy and radiation, although initially anti-tumorigenic due to direct tumor cell killing as well as release of immune-stimulatory antigens from dying cancer cells (6), can also exert pro-tumorigenic effects by causing release of pro-inflammatory cytokines by dying cancer cells with resulting expansion of immunosuppressive cell types (7). Checkpoint immunotherapies may be hindered by intratumoral T cell exhaustion (8). Therapies are needed that tip the balance between pro- and anti-tumorigenic effects and augment, not hinder, anti-tumor immunity in both the tumor microenvironment and in the periphery.

In order to manipulate the host immune system and take advantage of this powerful tool to eliminate tumors, a number of research areas and treatment approaches are being explored to enhance anti-tumor immunity. *Ex vivo* immune cell therapies, including adoptive T cell therapies and natural killer cell and dendritic cell based therapies, have potential to promote anti-tumor immunity and are effective in some types of cancer and hold promise for others (9). Synergies between chemotherapy or radiation (in which antigens are released) and dendritic cell based therapies (in which antigens are taken up and presented to T cells) should be explored; the sequencing and timing of these interventions will be prime considerations (9). Novel cancer vaccines, in which tumor neoantigens are elucidated from the patient’s tumor, given as an mRNA vaccine and then combined with current immunotherapy are being explored with promising preliminary results (10, 11). Finally, the gut microbiota affect the success of chemotherapy and immunotherapy by their modulation of the host immune system, as reviewed in (12), and dietary modification has been suggested to be an intervention that might improve the efficacy of immunotherapy (13).

In addition to the multipronged nature of its attack on cancer cells, another power of the immune system is its ability to produce a versatile response to clonal variation. It has been estimated that lung tumors comprise anywhere from 5-85% cancer cells, and the remaining cellular composition represents the host response (14). The cancer cells that comprise a tumor do not belong to a single clone, but consist of multiple different clones driven by a diverse set of somatic mutations as demonstrated by phylogenic analyses (15). Thus, the expectation that all cancer cells will be uniformly sensitive to a given therapeutic modality is unrealistic.

Target selection (typically based on differentially expressed or mutated proteins), drug design, and determination of mechanism of action are all critical features of drug development. However, it is not enough to demonstrate inhibition or reduction of tumor growth, and only focus on intrinsic tumor cell properties. How the compound drives the immune program should be incorporated into the study of any drug. The choice of an appropriate pre-clinical animal model is essential as the model should accurately identify the best drug candidates to move forward and then correlate with efficacy in humans.

## Considerations for clinical trial design and patient management

Designing appropriate clinical trials in oncology that account for the mechanism by which newer drugs work is essential if novel therapies, particularly ones that modulate the immune system, are to be incorporated into routine clinical practice. With recent advances in artificial intelligence/machine learning and *in silico* models, investigators will be able to more accurately predict the effect and outcome of a new cancer drug on diverse populations (16). This will help to optimize a number of features important for trial design including patient selection, sample size, monitoring criteria, and relevant endpoints. This will also lead to improved pharmacokinetic and pharmacodynamic modeling that can be used to optimize dosing schedules. These strategies should ultimately produce improvements in participation and reduce costs, while accelerating adoption of innovative therapeutic interventions. Additionally, they would allow for the ethical incorporation of novel immunotherapeutic agents as an early line of therapy when the ability of the immune system to respond is not compromised by multiple lines of prior therapy.

Currently, first line therapy almost exclusively represents standard of care while novel drugs with a unique mechanism of action are only offered once patients have failed conventional treatment. Since patients do worse with succeeding therapies, perhaps due in part to impairment of important elements of the immune system, testing new drugs in patients with fewer lines of therapy will be important.

Prior to treatment, in both clinical trial and patient management settings, the status of the immune system should be considered in addition to the genotype of the patient. Since the host needs a functional immune system to reject self-derived cancer cells, in essence an unusual form of autoimmunity, both innate and adaptive immunity are required. Patient evaluation with functional immune assays as well as for specific anti-tumor immunity at the time of diagnosis and throughout therapy would be beneficial. In addition, genetic polymorphisms and post-translational modifications relevant for effector function may be useful markers to pre-screen patients. Development of assays that evaluate immune competence and responsiveness is not a trivial exercise, but one that should be explored.

To change current treatment paradigms, several principles need to be reconsidered. First, if initial systemic therapy is given it should not cause immunosuppression that inhibits the necessary components that ultimately drive an anti-tumor host response. Second, the sequence of therapeutic interventions is important. Many tumors are resectable, whether for cure or debulking, and current thought suggests that delays may adversely affect survival. However, if the patient has not developed an adequate immune response against the tumor, initial surgical intervention in which tumor antigens and regional lymph nodes are removed could potentially limit antigen presentation, affinity maturation, and cross presentation required to drive anti-tumor immunity. Indeed, recent reports of several neo-adjuvant trials in early-stage lung cancer showing the efficacy of this approach supports this hypothesis (17, 18). Further attention to understanding the power of anti-tumor immunity could reap benefits in designing innovative protocols, particularly involving the preferred order of the various treatment modalities. In addition, immune profiling might be a more contemporary approach toward evaluation of drug efficacy, particularly compounds that work by modulating the immune system.

## Considerations for evaluating response to therapy

Finally, it will also be crucial to reconsider the metrics used to evaluate these drugs in routine practice and in clinical trials, and not always rely on size criteria by conventional imaging. Imaging provides detailed anatomic information, but the true cellular composition of a residual radiographic abnormality remains unknown. Prior studies have shown that initial radiographic response in patients with advanced stage disease does not consistently correlate with improved survival (19). Treatment may be stopped because tumors are stable in size or may even show initial progression, but this does not mean they will not ultimately inhibit tumor growth and metastasis. This is particularly true for drugs that work by modulating the host’s immune system, which may increase tumor size due to the inflammatory response. Better non-invasive imaging techniques that differentiate tumor cells from the host immune cell population are needed.

## Discussion

While immuno-oncology has been a prime focus over the past decade, we suggest that the field of medicine has not fully incorporated our understanding of anti-tumor immunity in treatment approaches and clinical trials design. To do this requires resources and innovation in order to create a paradigm shift that is necessary to improve the design of clinical trials for immunotherapeutic agents and eventually improve patient care. Disruptive technology in medicine is difficult to implement, but given the plethora of data to support the concept that driving host immunity will help control cancer, the time is now to rethink our approach to treating cancer patients. Many of the variables that influence patient outcome are not well understood, and we need to do a better job incorporating data from many different disciplines, so as to optimize a therapeutic approach that produces safer and better strategies with more durable improvements in outcomes.

## Data availability statement

The original contributions presented in the study are included in the article/supplementary material. Further inquiries can be directed to the corresponding author/s.

## Author contributions

EP: Conceptualization, Writing – original draft, Writing – review & editing. EG: Conceptualization, Writing – original draft, Writing – review & editing. GS: Conceptualization, Writing – original draft, Writing – review & editing.
